# Views and attitudes about the offer of NIPT: a qualitative study of UK healthcare professionals

**DOI:** 10.1186/s12910-025-01227-z

**Published:** 2025-07-19

**Authors:** Peter D. Young, Katherine M. Sahan

**Affiliations:** Ethox Centre Nuffield Department of Population Health, University of Oxford, Oxford, United Kingdom

**Keywords:** NIPT, Shared decision making, Non-directiveness, Prenatal screening

## Abstract

**Background:**

Healthcare professionals have ethical duties to provide information according to conceptions of the doctor-patient relationship, and one way this responsibility is established in practice is by UK guidance on shared decision making. Non-invasive prenatal testing (NIPT) is a relatively new prenatal screening test offered by the UK National Health Service (NHS) since 2021. Since NIPT has different characteristics when compared to other prenatal screens and tests—such as the combined test and amniocentesis—it is not clear how information should be offered in a pre-test consultation. Key to answering this question is to understand more about the HCP-patient relational dynamics surrounding the offer of NIPT. Previous studies have focused on the woman’s role in this; the views and attitudes of pregnant women about decision making in the offer of NIPT has been interrogated elsewhere. However, little attention has been given to the views and attitudes of healthcare professionals (HCPs) and how those views might shape the dynamics of how NIPT is offered and how the decision-making process goes.

**Methods:**

This study carried out qualitative interviews with 20 UK HCPs who offered NIPT and/or provided counselling for NIPT. Findings from the interviews were analysed and themes were developed about how HCPs reported they offered NIPT and their reasons for this.

**Results:**

HCPs say they conveyed information about the nature of NIPT to women when offering the test. This includes how HCPs say they described the risks of NIPT, their views about clarifying the non-diagnostic nature of NIPT, how they explained NIPT accuracy to women, and how they stressed that decisions about test options were up to the patient. HCPs also reported how they distinguished NIPT from other screens and tests and described NIPT as a different category of screening test. Furthermore, many HCPs say they either provided predetermined information to patients or reported being patient-led in the offer.

**Conclusions:**

This study explores how HCPs reported the offer of NIPT and also how they thought the offer should go, by giving their reasons for what they report. This indicates their normative sense of which information ought to be given (that is, what they believed was critical to provide for decision making). It also indicates which aspects within the offer they believed should be emphasised or played down. The accounts reported here of HCPs’ experiences raise questions about how information should be provided to women in the offer of NIPT. This might help us establish better practices of informing women who use NIPT. Results of this study have a number of implications for the ethics of prenatal testing in practice. Firstly, they indicate a need for better guidance and education about how to discuss certain informational aspects within the offer such as NIPT characteristics and statistics. Secondly, they show that aspects of the current offer may be value-laden, and the way HCPs counsel patients about NIPT may be insufficiently patient-led. More research in this area might tell us whether different guidance or educational opportunities ought to be developed to help HCPs discuss NIPT and its characteristics.

**Clinical trial number:**

Not applicable.

**Supplementary Information:**

The online version contains supplementary material available at 10.1186/s12910-025-01227-z.

## Background

### Responsibilities of HCPs when offering genetic tests

According to scholarship about the doctor-patient relationship, healthcare professionals (HCPs) have responsibilities to provide information to patients^1^ during decision making [1–3]. Approaches such as shared decision making (SDM) in the UK—a “collaborative process that involves a person and their healthcare professional working together to reach a joint decision about care”—are a way to uphold such responsibilities (4, para. 1). HCPs working with genetic technologies have additional commitments to work in a non-directive manner. Non-directiveness (ND) is generally understood as HCPs attempting to help patients “arrive at the best decisions from personal perspectives”, acknowledging the “critical importance of the patient’s wider value systems” [4, p. 135–6]. ND’s central purpose is to avoid “an easy confusion with, and moral contamination from, the eugenics movement” [4, p. 135], but also to uphold more general medical ethical principles such as respect for patient autonomy [5]. However, ND has been extensively critiqued, both because it might hinder the ability of HCPs to discharge their professional responsibilities adequately, and because in some cases, being insufficiently directive may thwart the best interests of patients themselves or their families [4]. SDM has been applied to the genetic medicine context in an attempt to build on the ND approach and strike the right balance between HCP and patient contributions to the decision-making process [4]. Such contributions are broadly construed as the balance between the offer^2^ of medically-informed evidence-based guidance (from the HCP), and specific perspectives, understandings, and preferences in relation to that offer (from the patient). Yet key challenges and empirical research questions remain in relation to how HCPs make such offers in practice. This is particularly so in the case of newer genetic tests and technologies such as NIPT entering the healthcare system, since HCPs will be developing their own understanding of how the test works. Therefore, their sense of what patients need to know and why they believe patients need to know it may not be firmly established. This is liable to exacerbate existing complexities in how to offer well or in the right way, balancing respect for patient choice against what HCPs feel is important to disclose.

### Prenatal screening tests in the UK

Currently, all pregnant women in the UK are offered free, first-tier screening tests through the National Health Service (NHS) for aneuploidies such as Down’s, Edwards’, and Patau’s syndromes (referred to as common trisomies here) [6]. One commonly used first-tier screen is the combined test, which is non-invasive and has low accuracy [6]. Therefore, results are not typically used to decide whether to continue or terminate a pregnancy. Instead, results can indicate high-risk pregnancies that could be offered further confirmatory testing for common trisomies [6].

When receiving a high-risk result for common trisomies via a first-tier screen, women can decide to have invasive, confirmatory testing in the NHS. Two invasive tests, chorionic villus sampling (CVS) and amniocentesis, are offered free of cost through the NHS to women with high-risk pregnancies [6]. Because of the physical invasiveness both tests, there is up to a 1% risk of procedure-related miscarriage associated with either test [6]. The results of these tests are diagnostic and can determine if the fetus has common trisomies [6].

### The introduction of NIPT and how it compares with other prenatal screens and tests

NIPT is a relatively new blood screening test that can be used to analyse cell-free DNA derived from the placenta and detected in the blood of pregnant women. It can be used to indicate whether a fetus might have a number of genetic anomalies [7, 8]. When there is a high enough concentration of cell-free DNA in the maternal bloodstream, usually by week 10 of the pregnancy [9], a maternal blood sample can be collected, and placental DNA can be isolated and sequenced [10]. Results indicate whether the fetus is at an increased risk for a genetic anomaly [8], although NIPT results are not diagnostic for common trisomies [10].

Since 2021, NIPT has been offered as a free, second-tier test throughout NHS England, and this offer is contingent on a positive test result from a first-tier test, such as the combined test. In the NHS, NIPT is primarily used to screen for common trisomies. Elsewhere, NIPT is also available through private UK clinics for an out-of-pocket fee paid by the user, which is not contingent on a first-tier positive test result. In addition to screening for common trisomies, some private UK clinics also offer NIPT screens for additional genetic anomalies.

**Table 1:** Characteristics of some screens and tests used to detect common trisomies in the UK

	First-tier screening	NIPT	Diagnostic tests
**Example**	Combined test	NIPT	Amniocentesis / CVS
**Materials / data which inform the test**	Pregnancy hormone levels and structural tests via ultrasound	Mixture of maternal DNA and cell-free DNA derived from the placenta	Amniotic fluid containing fetal cells / biopsy of placental tissue
**Invasiveness**	Non-invasive (e.g., blood draw)	Non-invasive (e.g., blood draw)	Invasive (e.g., long hollow needle inserted through abdominal wall / catheter inserted through the cervix)
**Risk to fetus**	No	No	< 1% chance of fetal loss
**Accuracy**	Low accuracy / Not diagnostic	High accuracy / Not diagnostic	Diagnostic
**Is it genetic based?**	No	Yes	Yes

Table 1 presents some of the similarities and differences between NIPT, first-tier screens, and diagnostic tests. NIPT and the combined test are both non-invasive screens. However, NIPT has higher accuracy than the combined test, and some women have wrongly perceived NIPT as diagnostic when testing for common trisomies [11]. Alternatively, invasive testing can provide diagnostic results for common trisomies, however, this comes at a very small risk of miscarriage. With NIPT, pregnant women can receive higher accuracy results, safely, and much earlier in the pregnancy—the combination of which is unique when compared to other prenatal tests for common trisomies [11, 12].

Additionally, other non-invasive screening tests used during pregnancy to screen for common trisomies are typically biochemical tests paired with ultrasound to indicate the likelihood of a fetus being affected by the condition. For example, the combined test checks for serum concentrations of two protein markers—pregnancy-associated plasma protein-A and human chorionic gonadotropin—and an ultrasound measures nuchal translucency and crown rump length. These parameters are considered with other factors to generate an assessment of how likely the fetus will have common trisomies based on a statistical algorithm. NIPT, however, is a genetic-based test, and this might change some of the background information that is provided with the test. When using NIPT, placental DNA—which in most cases is the same as fetal DNA—is isolated and analysed to see if the sequenced DNA indicates a genetic anomaly. The distinction of NIPT being a genetic-based screen versus other prenatal screens being biochemical- and structural-based screens is a key difference between both. In summary, NIPT is used as a screen for common trisomies in the NHS, is non-invasive, uses genetic information, and it is not paired with a functional test like an ultrasound. This different combination of characteristics makes it hard, especially in the context of other screening tests, to convey NIPT’s clinical significance. This could lead to complexity around how to present the offer of NIPT.

### Varying views on how the offer of NIPT should be presented

HCPs in the UK are educated about how to offer NIPT through training programmes and e-learning modules [13, 14]. The specifics of that training are unclear from the publicly available evidence. In addition to these training opportunities, there is guidance available that instructs HCPs on what information could be presented when offering NIPT, how to manage results, and how to understand other technical aspects of the test [15, 16]. However, that guidance does not discuss responsibilities, roles, and dynamics that surround the process of offering NIPT—for example, how to practise SDM [17]—in great detail. Similarly, the academic literature varies in how the offer of NIPT should be made, including what information should be given and the reasons for this. For example, some believed the offer could be made with limited information because there is no risk of physical harm to the pregnant woman or fetus when using NIPT [18], or to avoid informational overload, which risks lowering the quality of decision making [19]. Similarly, others believed setting informational limits might be appropriate if the stakes of testing are lower, for example, when NIPT is offered in private clinics without a first-tier positive test and so functions only as reassurance of an unaffected pregnancy [20].

However, the opposite stance is also found, with some commentators advocating for a more comprehensive disclosure of information within the offer to allow for more clarification and discussion which corrects for mistaken perceptions that NIPT is more like an invasive, diagnostic test [11, 21]. Correcting perceptions in this way is held to lessen the chance of poor decision making both about whether to have NIPT itself and future decisions that could arise from its results, for example, termination of pregnancy.

In summary, there are questions concerning how to offer prenatal genetic tests like NIPT which are set against a background of concerns. Firstly, how should HCPs make an offer which is sufficiently non-directive while upholding principles of SDM? Secondly, how is the offer adequately informative, conveying NIPT’s proper clinical significance in comparison with, for example, other diagnostic prenatal tests? Thirdly, what constitutes a ‘good’ offer which supports women well with decision making in the interests of themselves and their pregnancy? Given a dearth of qualitative studies focusing on how UK HCPs consider the offer of NIPT, this study uses in-depth, semi-structured interviews [22], to investigate the views of HCPs about the offer of NIPT.

## Methods

The overall project in which this study appears uses empirical bioethics as the research methodology [23]. This methodology uses philosophical analysis and empirical data collection to argue toward normative considerations. The study presented here comprises one of the empirical sections of that overall study, and the aim of this paper is to describe the views and attitudes about the offer of NIPT from HCPs. Therefore, 20 UK HCPs were interviewed in 2020 about the ethical and professional issues concerning the offer of NIPT.

The aim of this study was not to find a representative sample, but rather to collect a diversity of viewpoints from individuals working in the field. A recruitment process was undertaken that might access HCPs from different regions of the UK, from different professions, and from a variety of professional practice sites. Thus, participants were recruited by convenience, snowball, and purposive sampling methods. Prospective participants were contacted by email, however the number who refused to participate or the reasons why individuals refused to participate is unknown.

Of the 20 recruited participants, 12 were recruited from NHS clinics whilst 8 were recruited from private non-NHS-affiliated clinics. Their professions were sonography, genetic counselling, clinical genetics, midwifery, obstetrics and gynaecology, and healthcare counselling. The number of years of work experience in their respective roles ranged from 1 to 30 years with a median of 11 years. Seventeen of the participants were women and three were men. Table 2 presents a summary of study participants’ professions and their work settings, data which has been summarised elsewhere [24].

**Table 2:** Study participants by profession and work setting

Profession	Private	NHS	Total number
Obstetrician	2	2	4
Clinical geneticist	0	3	3
Genetic counsellor	1	3	4
Midwife	3	4	7
Sonographer	1	0	1
Healthcare counsellor	1	0	1
**Total**	**8**	**12**	**20**

All participants had experiences offering and/or providing counselling for NIPT in the UK. Whilst NIPT was introduced country-wide in NHS England in 2021, there were several NHS trusts in England that were already participating in a national NIPT rollout scheme, meaning some professionals were already offering NIPT in their workspaces at the time of interviews in 2020. The views expressed in interviews reflect participants from clinics that were already using NIPT in some capacity. In order to participate in this study, participants were required to have real experiences offering and/or providing counselling for NIPT in the UK.

A semi-structured interview guide was developed and used for this study (See supplementary file). Using that guide, interviews took place online in 2020. Informed consent to participate was obtained from all participants in the study. The length of interviews ranged from 45 min to 131 min with a median of 74 min. Findings from the interviews were analysed using themes that arose from select literature written about the doctor-patient relationship [1–3]. This work builds on top of a critical analysis of themes that arise in the doctor-patient relationship (unpublished manuscript). Some of the themes found through that analysis include the balance of harms and benefits and the exchange of facts and values. From there, initial codes were developed using those literature-based themes, and transcripts were read line-by-line with the initial codes being used to identify appropriate segments of text [25]. Secondary codes were then developed from the reading and analysis of the interview transcripts [25]. Once a list of secondary codes was defined, interview transcripts were re-read, and the new set of codes was applied to appropriate sections of text. From here, themes were developed from the collected dataset [26]. NVivo software was utilised in this process to manage data analysis. The collected dataset and analysis produced a rich description of the views and attitudes of HCPs in the offer of NIPT, and this current study looks at one aspect of that descriptive work—HCPs’ views and attitudes about how to offer NIPT to pregnant women. Themes are presented below, and representative quotations are listed underneath each theme.

## Results

### Theme 1– HCPs sought to convey key information about the nature of NIPT

When offering NIPT, many HCPs interviewed here said they highlighted information related to the conditions NIPT tested for, its non-invasiveness, the implications of test results, test accuracy, NIPT not being diagnostic, the differences between screening and diagnostic testing, false positives and false negatives, the possibility of a test failure, issues related to the timing of the test, and considerations related to test financing. Many of these points of information—i.e., what was tested for, the specific accuracy figures, test timing, and financing—were all things that could change depending on the specific pregnancy, clinic, laboratory service used, and whether the interviewee worked for an NHS clinic or a private one. The following quotations demonstrate some of the topics listed above.

*We felt it was important to give the full picture but be very clear what we could fund* [sic] *what we couldn’t fund* [sic] *and what were the potential limitations of non-invasive prenatal testing… Well*,* it’s not diagnostic. That’s obviously quite a key limitation. The test that we use only tests for trisomy 13*,* 18*,* and 21*,* so they’re not getting information about any other chromosomes*,* and we do not offer sex determination.*

*Interview 8, NHS screening coordinator*

*I think NIPT changes it slightly. It’s a longer part of the journey if the result comes back as a high-chance result… again*,* it’s about making sure that women are fully informed about the timescale and what they would be offered at each stage of the journey.*

*Interview 10, NHS deputy screening coordinator*

Other quotations provided further below will demonstrate other points of information from the list of topics above.

### Theme 2– HCPs varied in how they described the risks of NIPT

In discussing the risks and benefits of the test, some HCPs said they highlighted that the test has limited risk or no physical risk.

*We tend to talk to women*,* let them know that it is an option*,* and if they have a high-risk test*,* then just really compare the risks of invasive*,* and obviously*,* there aren’t really any risks with the non-invasive as such.*

*Interview 3, NHS genetic counsellor*

*I say that it’s got the advantage of being a non-invasive test and therefore*,* they’re not putting the pregnancy at any risk.*

*Interview 4, NHS genetic counsellor*

Others made the distinction between physical risk and informational risk and held the view that there was not any physical risk to the pregnancy but there might be some risk associated with test results.

*NIPT doesn’t carry any of those risks of miscarriage [as an invasive test carries]*,* but it carries the same risks [as invasive tests] of an unclear result or a failed result.*

*Interview 7, NHS obstetrician*

Other participants wished not to use the word risk at all because of an anti-disability connotation that they suggest the word communicates. For example, to suggest a pregnancy was at risk of a trisomy would, in their view, be to assume that there was something inherently wrong with that condition. Instead, some participants used language to denote the ‘chance’ of certain anomalies, and they were careful not to label test results as good or bad news.

*I think it’s really important that we use neutral terminology because we can’t assume that*,* that would be bad news for women*,* it just may be unexpected*,* but it’s not necessarily bad news*,* so we have to be very careful in our delivery*,* and then we ask them*,* we try to assess how they feel about that because for some women even if they had been told there’s a high-chance of Down’s syndrome*,* they may not wish to have a diagnostic test.*

*Interview 6, NHS midwife*

*By the way*,* the only other thing is that I still tend to use terms*,* mostly because it’s in my head*,* high-risk and low-risk when discussing with other healthcare professionals. But when we’re actually talking to patients*,* we talk about high-chance and low-chance. But it just slips back from a professional point of view.*

*Interview 12, Private obstetrician*

### Theme 3– HCPs said they stressed that decisions about test options were up to the patient

Most interviewees spoke about informing women of their full range of testing options, and several interviewees mentioned women could choose whatever options they like. The exact nature of testing options was not always described clearly in interviews. When options were described in interviews the term ‘option’ was understood a few different ways depending on the context and specific panel of conditions for which NIPT was used. Importantly here, in the context of common trisomies, five interviewees directly reported the options they provided to women after a positive first-tier screening test, which might typically precede NIPT. These options included [1] do nothing [2], have invasive, diagnostic testing, or [3] have NIPT.

*We make that very clear to women that we have no expectation of what their decision is going to be and so we usually list the options as #1 do nothing*,* #2 consider testing which will give a 100% answer which is invasive to the pregnancy*,* or #3*,* and at the moment*,* consider self-funding a non-invasive prenatal test.*^3^

*Interview 8, NHS screening coordinator*

In most instances, interviewees agreed that women should be able to choose whatever options they wish. The ability for women to say ‘no’ to different options and ‘to do nothing’ was stressed by many participants.

### Theme 4– NIPT was distinguished from other tests and described as a different category of test

Interviewees reported that in consultations with women, they described NIPT as different from other prenatal tests. HCPs compared first-tier testing with NIPT, describing NIPT as a screen that samples material or DNA that is more directly related to the fetus, rather than (as in first-tier testing) testing the woman’s levels of pregnancy hormones and structural measurements of the fetus.^4^

*I would also try to explain to them that we’re looking at very tiny fragments of cell-free fetal DNA*,* so it’s looking at that the sort of material of the baby rather than pregnancy hormones and numbers of incidence.*

*Interview 6, NHS midwife*

*I explain that in very basic terms but this other type of test called NIPT doesn’t look at what’s average because this is about you and your baby and frankly nobody else matters; it’s just you and your baby. What this test does is it looks at little fragments of your baby’s DNA that are there in your blood.*

*Interview 11, Private midwife*

Participants’ descriptions of NIPT appeared to emphasise that the test analyses material more personal to the woman via the analysis of their own fetus’ genetic DNA; in contrast, HCPs described other screening tests using general incidence numbers or averages. This pattern of emphasis seemed to suggest that NIPT results were perceived as superior. Other claims of test superiority were more overt, though HCPs reported they were careful to emphasise NIPT as a screening and not as a diagnostic test, and to omit language relating to diagnostic testing:

*With the patients*,* I don’t tend to use phrases like false positives or false negatives because it’s screening. It’s not really a false positive or a false negative, because that would imply a diagnostic result*,* so I don’t tend to use that type of phrasing… I do compare the NIPT quite a lot to the routine screening and say*,* you know*,* that’s quite good. This is better.*

*Interview 3, NHS genetic counsellor*

Still other HCPs reported they make a distinction between NIPT and other tests in consultation by describing NIPT as an ‘advanced’ and more sensitive screening test compared to other screening tests, although also emphasising that NIPT did not provide a diagnostic result.

*We’ll talk to them about the fact that it’s not a diagnostic test*,* but it’s an advanced screening test*,* and we’ll quote our lab statistics in terms of sensitivity or specificity*,* and that obviously*,* compared to the combined screening or the quadruple screening that they’re engaged with already*,* we’ll obviously explain to them that this is obviously a more sensitive test.*

*Interview 6, NHS midwife*

In summarising the quotations under this theme, the way in which some interviewees said they describe NIPT to women suggested a possible new category of prenatal test for trisomy—an advanced screening test.

### Theme 5– provision of information was predetermined by many HCPs and patient-led by others

Some HCPs indicated they pre-determined what information about NIPT to discuss and in what level of technical detail, without reference to the preferences of the patient in front of them. For example, one genetic counsellor’s view was that patients do not usually care about technical details therefore justifying their predetermined approach to use a basic, non-technical explanation for the majority of patients:

*In my counselling I just explain how the test works… what it tests for*,* tells you and won’t tell you*,* and what it means*,* and what you can do with the information. That would be the focus*,* you know. Genetics is very interesting*,* but the bulk of our patients don’t really care how the science of the test works.*

*Interview 3, NHS genetic counsellor*

Other HCPs reported a great level of technical detail in their explanations but similarly seemed to have pre-determined that this level of technical description was appropriate without reference to the patient in front of them.

*And then when we explain the test to women*,* what we generally say is that the placenta sheds fragments of DNA into their blood stream and that continues during the pregnancy.… We talk about the laboratory being able to identify an over representation of DNA coming from a specific chromosome which is how the lab is able to arrive at a likely prediction that the baby is affected with one of the chromosome conditions. And we also discuss the fact that because this*,* the laboratory will also be looking at DNA that’s come from the mother*,* that the group of people who cannot have this testing would be people who’ve had the condition. So*,* for example*,* we would describe how the over representation of chromosome 21 DNA would be seen in a person who had Down’s syndrome and therefore if they had Down’s syndrome*,* they wouldn’t be able to have this testing.*

*Interview 8, NHS screening coordinator*

Only some HCPs suggested a patient-led approach with regard to the provision of information, namely that they are sensitive to the patient in front of them and guided by her preferences and cues about how much and how technical the information provided could be. For example, one midwife reported that her approach was to let women and couples lead the consultation.

*So*,* it’s about not making assumptions… giving them very straightforward*,* no-nonsense information about their options and then really follow their lead and direction*,* because obviously you don’t know quite how they feel or how much they want to know or prepare. So*,* it’s very personal from woman to woman*,* couple to couple. And just empowering them with the correct knowledge and accurate knowledge that they can make the next decision.*

*Interview 6, NHS midwife*

### Theme 6– HCPs held views about clarifying the non-diagnostic nature of NIPT

Several HCPs reported they took special care to clarify NIPT was a screen and not a diagnostic test to avoid harmful misperceptions which were hard to counteract.

*I think the most important thing is you tell women it is not diagnostic.… it’s still screening*,* and it will give you a risk of*,* or chance of*,* it will not give you a definitive answer*,* because I think some women rely on it as a definitive answer*,* especially if it’s an increased risk… If you*,* for example*,* get an increased risk that comes back… a very knee-jerk reaction is the women want to say*,* ‘right*,* OK*,* if it has got Down’s syndrome*,* I don’t want to proceed with the pregnancy. I just want to have a termination’.*

*Interview 16, Private and NHS midwife*

A particular negative consequence of such misperceptions was cited as a reactive decision to terminate a pregnancy based on a screening result (indicating increased risk or chance of a trisomy) rather than a firm diagnosis (actual trisomy). This suggests HCPs believed some women might want to treat NIPT as more definitive or clinically significant than it actually is as a way to manage uncertainty or process risks.

Some HCPs attributed issues in clarifying NIPT as non-diagnostic to difficulties in communicating the risk or chance of an affected fetus.

*So*,* we talk about that your risk or your result may come back as unlikely to be affected or very low risk in the sense it’s 1 in 10*,*000*,* but your baby may still be in that percentage. That means the baby is affected. So*,* I think it is difficult to explain false positives and false negatives.*

*Interview 6, NHS midwife*

A few HCPs reported how they tried to address the issue of risk communication using specific language such as ‘likelihood’ versus definitive statements.

*If*,* however*,* you do that test… and they come back saying it looks very*,* very*,* very likely that baby is affected by this condition… you need to be clear that NIPT does not give a definite yes/no answer. Saying it is very*,* very*,* very likely is not the same as saying yes… So that’s how I present it to them.*

*Interview 11, Private midwife*

Even though most HCPs felt the need to distinguish NIPT as non-diagnostic, the view of a few HCPs was that NIPT results should be reported as providing firm, diagnostic-level information.

*For the trisomies 13*,* 18 and 21*,* the non-invasive prenatal testing and the invasive testing are equivalent in accuracy if you get an answer.… they are equivalent in accuracy*,* they are both 99.8% accurate in relation to diagnosis of the trisomy.… So*,* you would say*,* ‘NIPT shows there is a 99.9% chance that your baby has Down’s syndrome.’ So*,* for the purposes of this discussion*,* we will say*,* ‘your baby has Down’s syndrome.’*

*Interview 7, NHS obstetrician*

*And even clinicians too that we’ve come across were almost saying to women that ‘it’s as near diagnostic as damn it’.*

*Interview 19, Healthcare counsellor*

In short, HCPs varied in whether they felt NIPT should be clarified as non-diagnostic or not. Some professionals also seemed to believe the distinction between screening results and diagnostic results was difficult for women to understand in the context of NIPT.

### Theme 7– explaining NIPT accuracy to women

Among the reports of HCPs interviewed here, there was a pronounced focus on describing the accuracy of NIPT to women. Describing accuracy meant telling women about some of the statistical figures related to the test and it typically meant telling women about the high detection, sensitivity, and specificity rates of NIPT. Many HCPs reported the actual statistical figures they say they discuss with women.^5^

*The Down’s syndrome accuracy percent is 99*,* Edwards’ is 97 plus*,* and Patau’s is 87% plus. So*,* I just run through those numbers with them and check they’re kind of happy with that.*

*Interview 3, NHS genetic counsellor*

*I break it down and give them indiv—as a group it reports 99.9 risk detection. If you break it down individually*,* then it’s 99.9 for Down’s*,* I think it’s 97.3 for Edwards’*,* and 93.8 for Patau’s. So*,* I break it down individually—those are the rates—and that it will give you a combined risk of 99.9.*

*Interview 16, Private midwife*

One frequently mentioned accuracy-related number that appeared in a majority of interviews was a ‘detection rate’ of 99%. The 99% figure was used to as a reason to explain why NIPT was a better test when compared to the combined test.

*We would direct to the NIPT laboratory that we’re using at the moment*,* I think they’re approaching 99% accuracy for Down’s*,* and so we would try and talk a little bit about that and just really give them an idea of why this is considered a more advanced test.*

*Interview 6, NHS midwife*

*The reason for mentioning the 99 at all is pointing out the fact that 1 in 6 with Down’s syndrome are missed by the combined test*,* and it’s one of the reasons why the NIPT is a better test.*

*Interview 12, Private obstetrician*

The above obstetrician therefore provided a reason as to why providing the 99% figure could be important for women. For him, it was pertinent to mention the 99% NIPT detection rate as it compares to the 85% detection rate of the combined test. Therefore, he made the case that when comparing the combined test and NIPT, the 99% statistic for NIPT is the appropriate comparator.

In contrast, there were some HCPs who came out against using the 99% figure. One clinical geneticist had concerns about how NIPT accuracy had been described within clinics, and he was especially bothered by the 99% figure and select accuracy figures that were used to promote the test.

*As a measure of how good is this test at detecting Down’s syndrome or other autosomal trisomy*,* the accuracy of the test is completely*,* it’s a complete waste of time. But that’s the figure that most commercial people offering NIPT*,* that’s the figure they use. And that’s really hopeless.*

*Interview 2, NHS clinical geneticist*

Thus, some HCPs believed that the 99% accuracy figure was misleading. Whilst there were reasons provided by HCPs above about why the 99% statistical figure should not be provided in consultations, others admitted to using the 99% figure themselves.

## Discussion

The HCPs interviewed here held a diversity of attitudes about how the offer of NIPT should be made, including what information they thought was important to provide to women, the reasons or attitudes they hold when providing that information, and the optimal ways to convey the clinical significance of NIPT (Themes 1–7).

### NIPT as a superior screening test unwillingly misrepresents risk and uncertainty

HCPs reported describing NIPT as a type of genetic screening test that was different from (and superior to) first-tier screening (Theme 4), because it came without physical risks (Theme 2) and could provide more direct genetic information about the pregnancy as opposed to merely structural information and levels of biochemical markers (Theme 4). However, this could wrongly suggest that results are somehow more weighty or significant than other types of information in the prenatal screening context. In some cases, HCPs reported how results could be taken as diagnosing fetal trisomy rather than giving a risk or chance of trisomy, and this could result in poor decision making (Theme 6). This instinct, on the part of some HCPs, to reinterpret NIPT as providing diagnostic-level information and so avoiding harder topics of risk interpretation and uncertainty reflects accounts of the ethics of uncertainty in genomic medicine. These accounts caution against an unrealistic rhetoric of ‘test to reduce uncertainty’ and advocate for a more constructive approach to managing uncertainty in clinical genomic practice [27]. With this in mind, HCPs offering NIPT should perhaps be cautious about emphasising NIPT as a *genetic* test for fear of overinflating its perceived clinical significance and actionability. Rather, they should emphasise result uncertainty, giving due consideration to the social and psychological complexities of returning genetic test results [28]. This could include thinking of ways to reduce the negative connotations of uncertainty as well as managing the communication of risk and uncertainty well [27].

### Awareness of using value-laden statements is important

Reported descriptions or implications of NIPT as superior or ‘better’ (Themes 4, 6 and 7) contrasted with HCPs who indicated a ND approach to the offer of NIPT (Theme 3) and HCPs who thought describing NIPT evaluatively (e.g., 99% test accuracy led to NIPT being a better test) was misleading (Theme 7, Interview 2). This raises questions about the appropriateness of value-laden statements (or statements that might be taken as value-laden) within the offer. Some HCPs may have regarded their descriptions as conveying mere objective facts about NIPT because they regarded them as scientifically accurate (e.g., HCPs in Theme 7 who described NIPT as having a numerically higher accuracy or detection rate). However, such facts might take on the qualities of both facts and value-laden statements when presented within the offer in a similar manner as described by Hilary Putnam when he discusses the collapse of facts and values [29]. Even so, it is unclear whether it is wrong—as ND might suggest—for HCPs to advance value-laden statements or even opinions and recommendations with respect to the offer. This is not only because of the practical difficulties in separating facts from values or evaluative elements, but also because of HCPs needing to discharge their professional responsibilities well towards their patients. HCPs have professional responsibilities to disclose the information they think patients should know but upholding those responsibilities may include sharing their opinions or even recommendations. This is consistent with an account of SDM in the genetic context which stresses the importance of HCPs not ‘abandoning’ patients to the decision but offering support and even recommendations about testing (which patients may choose to uphold or veto) [4]. Therefore, what seems important for HCPs who offer NIPT is to (a) check that their own language and communication is routed in the evidence base (not merely their anecdotal experience or personal viewpoint), (b) be aware of how comparisons and numerical scientific ‘facts’ may be misunderstood without the proper clinical counselling, and (c) stress that any value-laden statements or recommendations should not dictate but supportively inform decision making by the patient. This underscores the need for more precision in the approach to language and contextualising comparisons when offering NIPT.

### NIPT was perceived by some HCPs as a diagnostic test

One particular issue that HCPs reported on was the difficulty with making the distinction between screening and diagnostic tests (Theme 6). Misunderstandings about whether NIPT could produce diagnostic results have been seen elsewhere in the peer-reviewed literature. For example, 15% of genetic counsellors stated that they had at least one patient who terminated her pregnancy after an NIPT result [30]. Authors of that study reported that women might misunderstand NIPT screening results—thinking they were diagnostic—and terminate the pregnancy without further diagnostic testing. This kind of misunderstanding, also reported in the wider literature [31], may not only be due to the complexities of managing uncertainty (see section above); it may also reflect a categorisation problem of NIPT among other prenatal tests. It is not clear how one should convey the offer of a test that falls just short of the criteria that would make it diagnostic.

There was another problematic feature related to whether HCPs themselves understood NIPT as non-diagnostic. For example, under Theme 7, at least two HCPs seemed to believe NIPT provided results of a diagnostic standard. This coincides with a study that showed that some HCPs believed NIPT results reached a diagnostic standard [11], even though this does not agree with the current scientific literature about NIPT accuracy. Other HCPs who cited the 99% NIPT test accuracy figure seemed either to share this belief or to equivocate about whether NIPT was non-diagnostic when speaking with women. This is concerning given a 2020 ruling from the UK Advertising Standards Authority which pronounced that the 99% figure commonly used in NIPT advertising should not be used in brochures and on company websites [32]. In this non-legal ruling, three companies were found to have misled customers by quoting 99% ‘detection rates’. Those companies used the 99% figure on their websites without an accompanying explanation as to what that figure indicated. If the 99% figure should not be used in commercial advertising, this suggests it should also be omitted when HCPs make the offer.

Yet such misleading statistics persist in public understandings of NIPT. In a qualitative survey of NIPT users in the United States, the most frequently mentioned reason that motivated NIPT use was the level of accuracy [33]. The survey results also suggested that women who used NIPT believed a 1% chance of the test not working was thought to be negligible. This suggests that prevailing public (mis)understandings might be hard to shift from the NIPT discourse. So, in the short to medium term it might be important for HCPs to (a) know about the current state of public understanding, acknowledging but sensitively correcting misunderstandings if this comes up in discussion, and (b) be clear to themselves and patients about why these misunderstandings are so persistent (e.g., commercial incentives, patients who are anxious and seeking more reassurance than NIPT can provide, and technical difficulties in explaining the differences between accuracy rates and diagnosis).

### Decision making about NIPT needs to be sufficiently patient-led

One final question remains when considering how to make the offer of NIPT in a pre-test consultation: this is how far to construct the offer by pre-selecting topics versus being more patient-led (Theme 5). One important aspect here is how closely HCPs should adhere to a patient-provider model where they describe predetermined NIPT information to women, and how far they should pursue a patient-led model of being responsive to patient preferences for, e.g., certain types and quantities of information. The SDM literature advocates moving between those two models based on a judgement about the specific needs of the woman [34]. For example, UK SDM guidance suggests patients should be offered (presumably pre-determined) ‘resources’ (e.g., a book, flyer, or app) in advance of a discussion but that these resources should encourage the patient to think in a patient-led or -centred way, focusing on “what matters to them” (4, Sect. 1.2 & 1.4). This guidance stops short of saying that what matters to HCPs—e.g., the idea of HCPs advancing opinions or recommendations [4]—is also germane to SDM. Nonetheless, SDM guidance does emphasise the value of discussion between HCP and patient ahead of a decision. Since a good discussion between stakeholders in the decision-making process would seem to involve the ability to advance and understand each other’s viewpoints, this might allow scope for at least some HCP views (relating to the evidence, carefully advanced, operating non-directively) to factor into patients’ decisions.

The *Montgomery Ruling* issued by the UK Supreme Court—which clarifies the requirements for provision of information in the consent process—supports this in two ways. First, it emphasises the requirement for engaged discussion, stating that “even those doctors who have less skill or inclination for communication, or who are more hurried, are obliged to pause and engage in the discussion which the law requires” [35]. Second, it sets a standard of information provision that centres upon what a reasonable person would want to know if in the position of the patient [36, 37]. Contained in this second point is both the need for HCPs to centre the patient but also generalise on information provision to the standard of the reasonable person. Given that NIPT and genetic testing generally are still considered as complex to understand [28], it could be entirely necessary to at least offer extensive, technical descriptions of NIPT to satisfy the reasonable person standard if this information is requested by the patient. Where extensive information provision is necessary to uphold the legal duty to the *Montgomery Ruling* and the model of patient-led decision making, we must improve upon the time-constrained context of the NIPT offer that others have described [38].

### Limitations

There are some limitations to consider when reading this manuscript. When analysing interview data, there was attention given to capturing attitudes about the information HCPs say they provide in consultations. It is possible, however, that HCPs might have spoken differently with an academic researcher than they would with a pregnant woman. Therefore, the conclusions arrived at were informed by interpretations of HCPs’ reported speech, and they should not be considered an accurate account of any clinical or healthcare context. Instead, the data provided here are subjective accounts of participants who provide NIPT, including what they remembered on the day of the interview and what information they were willing to share. Additionally, there was no second person involved in coding and no intercoder analysis. However, coding trees and results were discussed with colleagues in bioethics who research NIPT and the ethics of decision making. Also, one of the authors (KS) independently reviewed quotations from the results section.

## Conclusion

This paper discusses the responsibilities HCPs have when offering NIPT, which are set against a background of concerns. These concerns include how HCPs might make an offer which is sufficiently non-directive while upholding principles of shared decision making, and it also includes how to adequately offer information that conveys NIPT’s proper clinical significance in a manner that supports women in the decision-making process. To understand HCPs’ views about some of these concerns, this study used in-depth, semi-structured interviews to investigate the views of HCPs about the offer of NIPT. Results include the general information about NIPT that HCPs reported they provide to women in the offer, including how HCPs say they described the risks of NIPT, their views about clarifying the non-diagnostic nature of NIPT, how they explained NIPT accuracy to women, how they distinguished NIPT from other prenatal screens and tests, and how they stressed that decisions about test options were up to the patient. Furthermore, many HCPs say they provided predetermined information to patients in a manner that might be insufficiently patient-led. The results of this work give a clearer picture of how the NIPT offer is made, and these results led to considerations about how HCPs might communicate good information about NIPT in a way that does not misrepresent risk and uncertainty nor give the appearance that HCPs valued the test themselves. Importantly, this paper discusses the relationship dynamics that exist between HCPs and patients and how NIPT consultations can be sufficiently patient-led yet include scope for HCPs to inform women about the NIPT topics they find important. As a result, this work might be used to inform future studies about NIPT and might contribute to academic and policy discussions about the optimal ways of providing information to pregnant women regarding prenatal, genetic technologies.

## Electronic supplementary material

Below is the link to the electronic supplementary material.

Supplementary Material 1

## Data Availability

The datasets generated during and/or analysed during this study are available from the corresponding author on reasonable request.

## Footnotes

1 In this paper, we use the term patient at times, even though pregnant women are not always considered patients. Additionally, we acknowledge that not all pregnant people are women and that sometimes couples make decisions about pregnancies together.

2 By ‘offer’ here we mean the offer of the genetic test, in this case NIPT, by the HCP to the patient, namely what information is given by the HCP and how it is given or presented, including any weighting or emphasis (using numbers, statistics, or value terms such as ‘better’) that the HCP gives to certain informational aspects. By thinking of the offer in this way, we broadly separate it from how patients might independently further characterise the offer and from the subsequent decision about whether to have the test. This reflects the study focus on how HCPs consider the offer and its presentation. In practice, however, the making of the offer, patient re-characterisation, negotiation of the offer, and the decision will be an overlapping, iterated set of exchanges within the decision-making process.

3 At the time of the interview, the screening coordinator quoted above could offer NIPT through her NHS clinic, however that NIPT had to be paid out of pocket. This is what was meant where she states, “consider self-funding a non-invasive prenatal test”.

4 Maternal blood hormonal concentrations and structural measurements of the fetus via ultrasound are considered with other factors such as maternal age, weight, ethnicity, and smoking status and inserted into a statistical algorithm that can determine a patient-specific risk score for each trisomy condition. This score is given as a risk ratio, e.g., 1 in 250 risk, 1 in 100 risk.

5 These accuracy figures were typically internal to the test and could not provide any information in relationship to the specific woman’s pregnancy or result.

## Abbreviations

CVS: Chorionic villus sampling
DNA: Deoxyribonucleic acid
HCP: Healthcare professional
NHS: National health service
NIPT: Non-invasive prenatal testing
NPV: Negative predictive value
PPV: Positive predictive value
SDM: Shared decision making
UK: United Kingdom

## References

[1] Burke G. Ethics and medical decision-making. Prim Care. 1980;7(4):615–24. 6908085

[2] Emanuel EJ, Emanuel LL. Four models of the physician-patient relationship. JAMA. 1992;267(16):2221–6. 1556799

[3] Veatch RM. Models for ethical medicine in a revolutionary age. Hastings Cent Rep. 1972;2(3):5–7. 4679693

[4] Elwyn G, Gray J, Clarke A. Shared decision making and non-directiveness in genetic counselling. J Med Genet. 2000;37(2):135–8. 10662816 10.1136/jmg.37.2.135 PMC1734519

[5] Harper P. Practical genetic counselling. 7th ed. London: Hodder Arnold; 2010.

[6] National Health Service. Screening for Down’s syndrome, Edwards’ syndrome and Patau’s syndrome. UK: National Health Service. 2018 [updated 2018; cited 2019 Nov 13]. Available from: https://www.nhs.uk/conditions/pregnancy-and-baby/screening-amniocentesis-downs-syndrome/

[7] Lo YMD, Corbetta N, Chamberlain PF, Rai V, Sargent IL, Redman CW, et al. Presence of fetal DNA in maternal plasma and serum. Lancet. 1997;350(9076):485–7. 9274585 10.1016/S0140-6736(97)02174-0

[8] Wright CF, Burton H. The use of cell-free fetal nucleic acids in maternal blood for non-invasive prenatal diagnosis. Hum Reprod Update. 2009;15(1):139–51. 18945714 10.1093/humupd/dmn047

[9] Chiu RWK, Lo YMD. Non-invasive prenatal diagnosis by fetal nucleic acid analysis in maternal plasma: the coming of age. Seminars Fetal Neonatal Med. 2011;16(2):88–93. 10.1016/j.siny.2010.10.003 21075065

[10] Norton ME, Jacobsson B, Swamy GK, Laurent LC, Ranzini AC, Brar H, et al. Cell-free DNA analysis for noninvasive examination of trisomy. N Engl J Med. 2015;372(17):1589–97. 25830321 10.1056/NEJMoa1407349

[11] Vanstone M, Yacoub K, Giacomini M, Hulan D, McDonald S. Women’s experiences of publicly funded non-invasive prenatal testing in Ontario, Canada: considerations for health technology policy-making. Qual Health Res. 2015;25(8):1069–84. 26063605 10.1177/1049732315589745

[12] Haidar H, Dupras C, Ravitsky V. Non-invasive prenatal testing: review of ethical, legal and social implications. BioethiqueOnline. 2016;5:6.

[13] Powell L, PHE Screening Blog. Hundreds have already attended new NIPT training. 2017 Nov 30 [cited 2025 Feb 10]. Available from: https://phescreening.blog.gov.uk/2017/11/30/hundreds-have-already-attended-new-nipt-training/

[14] Powell L, PHE Screening Blog. New NIPT e-learning resource live. 2020 Oct 23 [cited 2025 Feb 10]. Available from: https://phescreening.blog.gov.uk/2020/10/23/new-nipt-e-learning-resource-live/

[15] NHS England. Screening for Down’s syndrome, Edwards’ syndrome and Patau’s syndrome: non-invasive prenatal testing (NIPT). GOV.UK. 2024 Oct 8 [cited 2025 Feb 10]. Available from: https://www.gov.uk/government/publications/screening-for-downs-syndrome-edwards-syndrome-and-pataus-syndrome-non-invasive-prenatal-testing-nipt/e71987f8-3306-45d6-9dda-c0b6625ee7ed

[16] NHS England. Fetal anomaly screening programme handbook, GOV.UK. 2024 Dec 16 [cited 2025 Feb 10]. Available from: https://www.gov.uk/government/publications/fetal-anomaly-screening-programme-handbook/289099d7-ded0-43be-a901-600b78fb727e#non-invasive-prenatal-testing-nipt

[17] National Institute for Health and Care Excellence (NICE). Shared decision making. London: NICE. 2021 [cited 2025 Mar 10]. Available from: https://www.nice.org.uk/guidance/ng197/chapter/Recommendations 34339147

[18] van Den Heuvel A, Chitty L, Dormandy E, Newson A, Deans Z, Attwood S, et al. Will the introduction of non-invasive prenatal diagnostic testing erode informed choices? An experimental study of health care professionals. Patient Educ Couns. 2010;78(1):24–8. 19560305 10.1016/j.pec.2009.05.014

[19] Farrell RM, Mercer MB, Agatisa PK, Smith MB, Philipson E. It’s more than a blood test: patients’ perspectives on noninvasive prenatal testing. J Clin Med. 2014;3(2):614–31. 26237393 10.3390/jcm3020614 PMC4449684

[20] Ngan O, Yi H, Ahmed S. Service provision of non-invasive prenatal testing for down syndrome in public and private healthcare sectors: a qualitative study with obstetric providers. BMC Health Serv Res. 2018;18(1). 10.1186/s12913-018-3540-9 PMC6150999 30241520

[21] Lewis C, Hill M, Chitty LS. Women’s experiences and preferences for service delivery of non-invasive prenatal testing for aneuploidy in a public health setting: a mixed methods study. PLoS ONE. 2016;11(4):e0153147. 27045195 10.1371/journal.pone.0153147 PMC4821600

[22] Sandelowski M. Telling stories: narrative approaches in qualitative research. J Nurs Scholarsh. 1991;23(3):161–6. 10.1111/j.1547-5069.1991.tb00662.x 1916857

[23] Ives J, Dunn M, Cribb A. Empirical bioethics: theoretical and practical perspectives. Cambridge: Cambridge University Press; 2018.

[24] Young PD. Attitudes about NIPT routinisation: a report from a qualitative study of 20 UK healthcare professionals. Health Care Anal. 2025. 10.1007/s10728-025-00513-6 10.1007/s10728-025-00513-6 PMC13675319 39982639

[25] Yin RK. Qualitative research from start to finish. London: Guilford Press; 2011.

[26] Braun V, Clarke V. Thematic analysis: A practical guide. Los Angeles: SAGE; 2021.

[27] Newson AJ, Leonard SJ, Hall A, et al. Known unknowns: Building an ethics of uncertainty into genomic medicine. BMC Med Genomics. 2016;9:57. 27586379 10.1186/s12920-016-0219-0 PMC5009566

[28] Geller G, Botkin J, Green M, Press N, Biesecker B, Wilfond B, et al. Genetic testing for susceptibility to adult-onset cancer: the process and content of informed consent. JAMA. 1997;277(18):1467–74. 9145720

[29] Putnam H. The collapse of the fact/value dichotomy and other essays. Cambridge: Harvard University Press; 2004.

[30] Buchanan A, Sachs A, Toler T, Tsipis J. NIPT: current utilization and implications for the future of prenatal genetic counseling. Prenatal Diagn. 2014;34(9):850–7. 10.1002/pd.4382 24711206

[31] Howard HC, Borry P. Personal genome testing: do you know what you are buying? Am J Bioeth. 2009;9(6–7):11–3. 10.1080/15265160902894005 19998102

[32] Advertising Standards Authority. Non-Invasive Prenatal Testing (NIPT)– A look at the ASA’s rulings. 2020. Available from: https://www.asa.org.uk/news/non-invasive-prenatal-testing-nipt-a-look-at-the-asa-s-rulings.html

[33] McDowell M. Making sense of numbers about health risks—The facts box. In: Elwyn G, Edwards A, Thompson R, editors. Shared decision making in health care: achieving evidence-based patient choice. Oxford: Oxford University Press; 2016. pp. 123–8.

[34] National Institute for Health and Care Excellence (NICE). Shared decision making. London: NICE. 2021 [cited 2025 Mar 10]. Available from: https://www.nice.org.uk/guidance/ng197/chapter/Recommendations. Point 1.2.5. 34339147

[35] [2015]. UKSC 11, issued by the UK Supreme Court in April 2015. Para 93.

[36] [2015]. UKSC 11, issued by the UK Supreme Court in April 2015.

[37] Herring J, Fulford K, Dunn M, Handa A. Elbow room for best practice? Montgomery, patients’ values, and balanced decision-making in person-centred clinical care. Med Law Rev. 2017;25(4):582–603. 28985348 10.1093/medlaw/fwx029

[38] Bowman-Smart H, Perrot A, Horn R. Supporting patient decision-making in non-invasive prenatal testing: a comparative study of professional values and practices in England and France. BMC Med Ethics. 2024;25:34. 38515078 10.1186/s12910-024-01032-0 PMC10956335

