# Supplementary material for: Views and attitudes about the offer of NIPT: a qualitative study of UK healthcare professionals
Source: BMC Med Ethics. 2025 Jul 19;26:101. doi: 10.1186/s12910-025-01227-z (PMC12275380; doi:10.1186/s12910-025-01227-z)
Supplement: Supplementary file 1 — Supplementary Material 1 [file 12910_2025_1227_MOESM1_ESM.pdf]

Views and attitudes about the offer of NIPT:
A qualitative study of UK healthcare professionals

Supplementary file – Interview topic guide

Opening questions

1. As I understand it, you are a [interviewee's profession]. Is that correct?
2. How many years of experience do you have as a [interviewee's profession]?
3. What is your role (on your ward or) in your professional setting?
4. How long have you been offering prenatal screening tests?
5. What is your involvement with prenatal tests?
6. Can you tell me more about your experience of offering prenatal screening?

Offering NIPT

7. How often do you discuss NIPT with women?
8. Do you offer it to all women?
9. How do you introduce NIPT to women? What do you tell them?
10. Is there anything you think is important to discuss with women prior to offering prenatal testing? Does this change in the offer of NIPT?
11. How do you present the advantages and disadvantages of the test, if you think there are any?
12. How do you feel about the offer of NIPT? What, if anything, would you like to see changed about how NIPT is offered?

Return of results

13. Who returns NIPT results to women in your clinic?
14. Can you describe how results are communicated to women?
15. What do you think is important when returning results?

Post-test counselling

16. Once test results are returned, how do you support women in decision-making about their pregnancy?
17. How do you feel about the support offered to women to make their own decisions about their pregnancy? What, if anything, would you change?

Reflection / closing questions

18. Do you feel there are any other ethical issues related to NIPT that we have not yet discussed?
19. How are your experiences with NIPT similar to or different from other prenatal tests that are currently available?
20. Is there anything else you would like to discuss with regards to NIPT?
